# First person – Sarah Alghamdi

**DOI:** 10.1242/dmm.049730

**Published:** 2022-08-03

## Abstract

First Person is a series of interviews with the first authors of a selection of papers published in Disease Models & Mechanisms, helping early-career researchers promote themselves alongside their papers. Sarah Alghamdi is first author on ‘ Contribution of model organism phenotypes to the computational identification of human disease genes’, published in DMM. Sarah is a PhD student in the lab of Robert Hoehndorf at King Abdullah University of Science and Technology, Thuwal, Saudi Arabia, investigating artificial intelligence, specifically knowledge representation and reasoning over biomedical data.


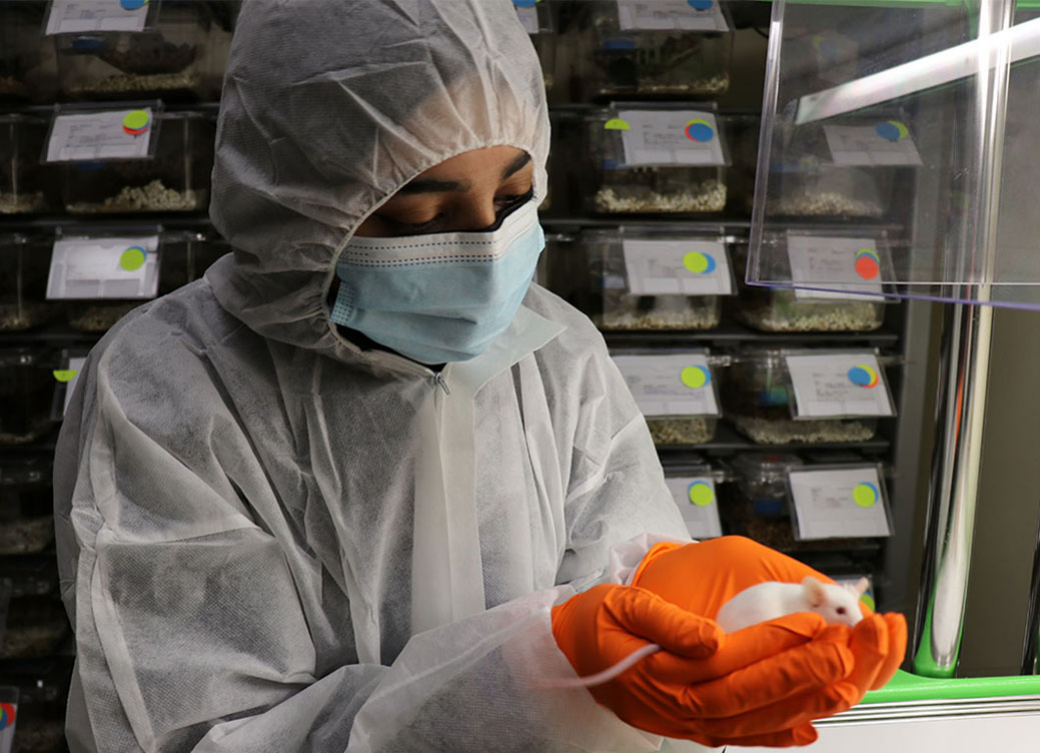


**Sarah Alghamdi**

**How would you explain the main findings of your paper to non-scientific family and friends?**

Model organisms are non-human organisms – such as the mouse, fish, fruit fly and yeast – that are intensively studied and can give us insights into human biology, including identification of disease-causing genes and the underlying mechanisms. One approach to finding human disease genes is to test whether genetic perturbations in the organism lead to phenotypes that look or behave similarly to those of a human with the disease under investigation. This is usually difficult due to the intrinsic differences between organisms, but phenotype ontologies – methods that systematically relate characteristics of humans and model organisms to each other – capture the similarities between phenotypes across species and allow us to compare them. Using a spectrum of methods devised by us and others to identify disease genes through phenotype comparison, we investigated how these methods performed and how much each species' data contributes to disease gene discovery. We identified several biases in the underlying data – for example, genes that are associated with a disease in humans are generally studied more thoroughly in model organisms. When correcting for these biases, the mouse shows the best and most computationally useful similarity to human phenotypes, while fish, fruit fly and yeast do not contribute significantly.

“[…] the mouse shows the best and most computationally useful similarity to human phenotypes […]”

**What are the potential implications of these results for your field of research?**

Our results show that non-mammalian phenotype–genotype data are not helpful in human disease gene identification using existing algorithms, and indicate that we need to develop new artificial intelligence methods relating model organism phenotype data to human disease. We also see intrinsic biases in the data in model organism databases and annotation strategies. The machine learning methods we tested are very sensitive to several biases that we have identified and explored, and I hope my work allows other researchers to develop and employ methods to correct for these biases. In addition, our research can inform the collection and annotation of new data and in particular improve annotation guidelines so that biases are avoided or at least made explicit.

**What has surprised you the most while conducting your research?**

During my analysis I used several machine learning methods, and, surprisingly, in each of the methods I used, I was able to achieve good predictive results, even when using yeast phenotypes in predicting human gene–disease associations. Yeast phenotypes were able to correctly identify gene–disease associations, even when the disease was complex and involved things like anatomical development, behavioural changes, etc. My co-authors were sceptical about this result so I spent a lot of time digging through the data and trying to find what might be going wrong – did I make a mistake or is there something strange going on with the data? After analysing several hypotheses, I identified different biases in the underlying data. When correcting for these biases, only the mouse shows good prediction of human disease genes. Understanding and characterizing these biases now makes up a large part of my paper.

**Figure:**
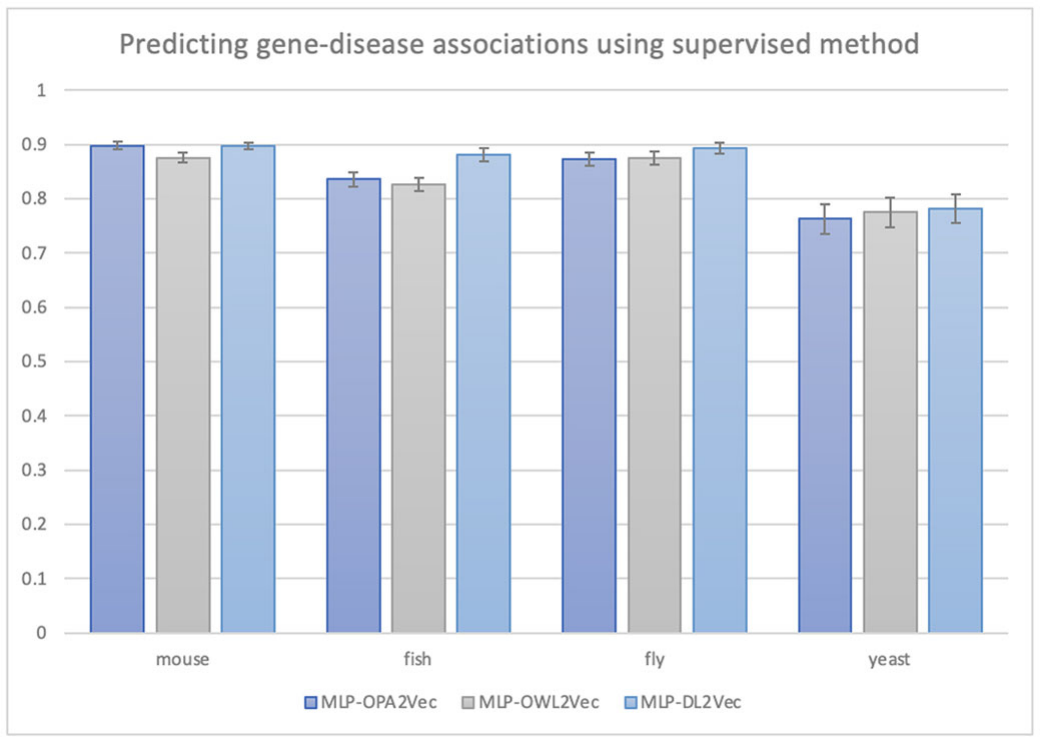
**Gene–disease association prediction results using supervised learning.** The results demonstrate that the underlying data contain substantial bias that can be exploited by machine learning models.

**Describe what you think is the most significant challenge impacting your research at this time and how will this be addressed over the next 10 years?**

In this context, two primary challenges can be discussed. First is the development of model organism research as a tool for gene discovery and phenotype collection in rare disease. This task requires a substantial knowledge of model organism biology and genetics in order to tailor the experimental design and analysis to a particular gene. This process also requires the hiring of PhD-level scientists capable of conducting research in a sustainable manner. Then, following the collection of data from model organisms, the problem of relating the phenotypes of different species using formal ontologies arises. In principle systematic collection of phenotype data, such as is done in the International Mouse Phenotyping Consortium, would help with some of the biases caused by using hypothesis-driven research results, but the vast bulk of the very rich data we have is still uniquely valuable. The ontology evaluation step for phenotype ontologies is a fundamentally difficult task, and we continue to observe unintended inferences being drawn from phenotype ontologies. The difficulty arises when reasoning algorithms fail to detect incorrect inference and we need to address these methodologies, possibly even looking at non-ontology dependent methods.

**What changes do you think could improve the professional lives of early-career scientists?**

I was fortunate to study at King Abdullah University of Science and Technology, which offers a family-friendly environment, with healthcare and childcare provided for students, researchers and their families. Many students and early-career scientists I know have given up on their careers due to financial pressure and the challenge to balance family life and their work. As research is a very competitive career path, I believe that universities and research institutes should increase the financial support level for researchers and students just to not lose ambitious young scientists. As the mother of two young pre-schoolers, I could not have accomplished any of my work without financial, healthcare and childcare support.

“Many students and early-career scientists I know have given up on their careers due to financial pressure and the challenge to balance family life and their work.”

**What's next for you?**

Phenotype data are important for understanding the biological mechanisms behind a disease. Through the use of ontologies, researchers were able to formally characterize and classify phenotypes, thereby aiding in the identification and interpretation of disease mechanisms. In my country, consanguineous marriage is the most important reason for the development of rare diseases. Saudi Arabia has the highest autosomal recessive birth rate in the world, according to a 2022 study. It remains difficult to identify the genes and variants responsible for such rare diseases. My work on phenotypes and phenotype similarity contributes to finding disease-associated genes and variants. Therefore, I will continue to work on improving the ontological phenotype representations, and the methods using them in finding disease-associated genes and variants, so that I can contribute to solving the challenge of rare diseases in my country.
